# Halotolerant Mycorrhizal Symbiosis Enhances Tolerance in *Limonium* Species Under Long-Term Salinity

**DOI:** 10.3390/genes16091084

**Published:** 2025-09-15

**Authors:** Catarina Gomes-Domingues, Isabel Marques, Maria Cristina Simões Costa, Ana D. Caperta

**Affiliations:** 1 Linking Landscape, Environment, Agriculture and Food (LEAF) Research Center, Associate Laboratory TERRA, Instituto Superior de Agronomia (ISA), Universidade de Lisboa, Tapada da Ajuda, 1349-017 Lisboa, Portugal; catarinaagomes01@gmail.com (C.G.-D.); simoescosta@isa.ulisboa.pt (M.C.S.C.); 2 Forest Research Centre (CEF), Associate Laboratory TERRA, School of Agriculture (ISA), University of Lisbon, Tapada da Ajuda, 1349-017 Lisbon, Portugal; isabelmarques@isa.ulisboa.pt

**Keywords:** chlorophyll fluorescence, gene expression, halophytes, *Limonium* spp., salt glands, salt tolerance, stomatal conductance

## Abstract

To survive in saline environments, plants establish complex symbiotic relationships with soil microorganisms, including halotolerant arbuscular mycorrhizal fungi (AMF). The main objective of this study was to uncover how inoculation with a consortium of halotolerant AMF influences recretohalophyte *Limonium* species tolerance to long-term salinity, at physiological and molecular levels. In this study, the physiological performance, ultrastructure of leaf epidermal cells, and expression of seven genes involved in salinity response were studied in *Limonium daveaui* and *Limonium algarvense* plants exposed to 200 mM NaCl and inoculated with an AMF consortium, dominated by *Rhizoglomus invernaius*. An isohydric response was observed for both species after one year in salinity. Inoculation with AMF led to higher stomatal conductance for plants in non-saline conditions and improved photosystem II efficiency under salinity. In *L. algarvense*, inoculation enhanced stomata and salt gland epidermal area under tap water. While salinity significantly increased salt gland, stomata and pavement cells areas but not cell size. In *L. daveaui*, AMF led to an increased salt gland density as well as salt gland size under saline conditions. In both species, salinity increased the expression of Na^+^/H^+^ antiporter *AtSOS1*, aquaporin *TIP5*, and salt gland development related genes *LbTRY*, *Lb7G34824* and *Lb4G22721GIS2*. The expression of such genes was significantly reduced in AMF-inoculated plants under salinity. Besides, higher levels of gene expression were observed in *L. algarvense* than in *L. daveaui*. Overall, our findings highlight the protective role of halotolerant AMF and emphasize their potential as sustainable effective bio-inoculants for enhancing plant salinity tolerance.

## 1. Introduction

Land salinization is a process affecting people and ecosystems throughout the planet and is currently recognized as a main threat to agriculture and human health [1,2]. In arid and semi-arid Mediterranean environments, soil salinity may result from either natural process, such as capillary rise of saline groundwater or seawater intrusion in low-lying coastal areas, or anthropogenic activities, including improper irrigation techniques, inadequate drainage, and overextraction of groundwater [3,4]. Understanding these processes is critical for breeding or engineering crops with improved salt tolerance.

Salinity stress is one of the most dangerous threats to agriculture causing plant vegetative and reproductive growth inhibition, limiting crop productivity, and leading to substantial yield reduction in most plants [5,6]. Among the primary mechanisms, salinity imposes osmotic stress, due to the presence of water soluble salts, reducing soil water potential and limiting water and nutrient uptake, mimicking drought-like conditions even when water is physically present [2,7]. This “physiological drought” leads to reduced cell expansion, stomatal closure, and impaired photosynthesis. Simultaneously, ion toxicity occurs, primarily due to the accumulation of Na^+^ and Cl^−^ in the older tissues which can disrupt cellular homeostasis, enzyme activity, and membrane integrity. Moreover, salinity triggers secondary stress responses, including oxidative stress, due to the overproduction of reactive oxygen species (ROS) such as hydrogen peroxide and superoxide radicals [8,9,10]. If not adequately scavenged, these ROS can damage lipids, proteins, and nucleic acids, leading to cellular dysfunction and death. The severity of these responses can vary greatly depending on species, genotype, developmental stage, and environmental context.

While glycophytes (salt-sensitive, most crops) are highly sensitive to those effects, halophytes (salt tolerant) offer a contrasting model of resilience. Halophytes are extremophile plants capable of surviving and completing their life cycle in environments containing high concentrations of soluble sodium salts (80 mM NaCl, [11]; 200 mM NaCl, [12]). Besides, halophytes exhibit optimal growth under specific salinity levels, with high NaCl concentrations frequently promoting their development, biomass accumulation and overall plant vigor [8,13,14]. Thus, they have evolved specialized morphological, physiological, and molecular adaptations to cope with high salinity, including efficient ion compartmentalization in vacuoles, active exclusion of toxic ions at the root level, accumulation of compatible solutes, and robust antioxidant systems. These adaptations make halophytes sustainable alternatives in salt affected lands under saline conditions that inhibit glycophytes growth.

Another remarkable survival strategy, adopted by both glycophytes and halophytes under stress conditions, is the association with different soil microorganisms. Particularly, in saline environments plants are colonized by halotolerant arbuscular mycorrhizal fungi (AMF) which have evolved to adapt to different salinity conditions [14,15,16,17]. Mycorrhizae have been shown to confer many beneficial effects to plants. For instances, AMF inoculated plants possess a higher root development, improving plant–water status and plant nutrition (higher K/Na and Ca/Na ratios in plant tissues) [15,16,17,18,19,20]. Mycorrhizae are also described to enhance chlorophyll and carotenoid synthesis, stomatal conductance, transpiration and photosynthetic rates, improving plant growth and performance. Additionally, they also prevent element toxicity, improve osmotic adjustment and protection against oxidative damage and overall enhancing plant tolerance to salt stress [15,16,17,18,19,20]. Finally, AMF differentially regulate the expression of several plant genes (e.g., aquaporin genes; genes involved in proline biosynthesis) in organs of plants exposed to salinity [18,21]. The use of salinity adapted AMF can be employed as a potential sustainable strategy to protect plants against salt stress and improve plant growth in salt-effected soils [16,20].

Salt tolerance is a complex trait controlled by multiple genes, that influence a wide range of physiological and biochemical processes [7,22]. This trait results from the cumulative effects of many loci, often with small individual contributions but complex epistatic interactions. Loci linked to components of salt tolerance have been identified in various glycophyte species [23,24]. Still, halophyte species are increasingly utilized as model systems for dissecting the molecular basis of salt tolerance, particularly through the characterization of salt stress-responsive genes, transcriptional regulators, and signalling pathways [13,14]. The key halophytic genes related with salt tolerance include, among others, vacuolar membrane-localized cation/H^+^ antiporters, pyrophosphatases, plasma membrane-associated transporters, particularly components of the Salt Overly Sensitive (SOS) signalling pathway, which play a crucial role in regulating Na+ influx and overall salt stress response [13,14]. This includes SOS1 (a plasma membrane Na^+^/H^+^ antiporter) SOS2 (a protein kinase), and SOS3 (a calcium-binding protein), that function to expel Na^+^ from root cells and maintain ion balance under stress. Moreover, tonoplast intrinsic protein (*TIP*) genes encode membrane proteins belonging to a subfamily of aquaporins that regulate water movement and contribute to maintaining osmotic adjustment under saline conditions [25].

In the Plumbaginaceae family, multicellular salt glands present on the epidermis of their aboveground tissues have been described in nine genera [26,27,28]. Those salt-secreting structures can secrete excessive ions like Na^+^, K^+^, Ca^2+^, Mg^2+^, Zn^2+^, Cl^−^, HCO_3_^−^, and SO_4_^2−^, with Na^+^ and Cl^−^ fluxes exceeding 200 pmol/gland/h [28,29,30]. In *Limonium* salt-secreting halophytes (recretohalophytes), these salt glands constitute a unique epidermal feature, complementing other well-studied epidermal cell types in land plants such as stomata [31], trichomes [32], and pavement cells [33], being the first structures involved in the regulation of salt tolerance [8,26,27,28]. For instances, *Limonium algarvense* secretes salt through a complex 16-celled salt-gland structure in the leaf epidermis. This species colonizes sandy well drained soils inundated by the sea water during high tides [34]. Another example is *Limonium daveaui*, a recretohalophyte also occurring on thermomediterranean dry saltmarshes and adapted to extreme summer drying and salt efflorescence [35]. Recent investigations in *Limonium bicolor*, using single-cell RNA sequencing of protoplasts from young leaves, have identified molecular determinants potentially involved in the regulation of salt tolerance [36,37]. Among them, the *TRIPTYCHON* (*LbTRY*) gene and its interacting protein Lb7G34824 coregulate salt gland development. By combining with GLABROUS INFLORESCENCE STEMS 2 (*LbGIS2*) gene, *LbTRY* also regulates cytokinin signal pathway, helping to keep cytokinin homeostasis and stabilize salt gland development [37]. Understanding the strategies employed by halophytes provides valuable insights into the mechanisms of salt tolerance.

Previously, positive effects of non-native AMF have been reported on the performance of *L. algarvense* under saline conditions [38]. Besides, the use of AMF native from a saline environment results in beneficial aids for *Limonium* plants [39]. This study aims to characterize the physiological performance and gene expression patterns in leaves of two *Limonium* species under prolonged saline conditions at 200 mM NaCl. The main objective was to uncover how inoculation with a previously characterized consortium of halotolerant AMF, dominated by *Rhizoglomus invernaius*, influences stress responses at the molecular level, by identifying differentially expressed genes involved in salinity adaptation. The specific goals of this study were to: (1) assess the physiological performance of *Limonium* plants with and without mycorrhizal inoculation; (2) examine the ultrastructure of leaf epidermal cells; (3) quantify the relative expression of a set of target genes implicated in salt tolerance mechanisms in *Limonium*; and (4) contextualize the results to elucidate the potential protective role of halotolerant arbuscular mycorrhizal fungi in enhancing plants salinity tolerance.

## 2. Materials and Methods

### 2.1. Plant Material and Experimental Set Up

In this experiment, twenty plants of each species, *L. daveaui* and *L. algarvense*, were grown under greenhouse-controlled conditions at the Instituto Superior de Agronomia (ISA), University of Lisbon, Portugal. Plants originated from seeds collected from natural populations in saltmarshes in Fundação do Samouco salterns complex (Alcochete, Portugal) and in Guadiana estuary (Algarve, Portugal), respectively, and germinated as described in [40]. Briefly, after germination on water-soaked filter paper and growth in *jiffy* pots (peat) under controlled conditions in a growth chamber, the three-month-old seedlings were transplanted into plastic pots with a mixture of autoclave-sterilized (1 h at 120 °C) peat and perlite (1:2 *v*/*v*). The substrates of half of the plants were inoculated with a halotolerant AMF consortium (gently given by A. Nogales, Institute of Agrifood Research and Technology, Spain) isolated from the rhizosphere of saltmarsh *L. daveaui* plants, and root colonization was confirmed as described in [38]. This consortium was dominated by *Rhizophagus invermaius*, followed by an undescribed member of Glomeromycota, and other low-abundance species, including *Funneliformis* sp., *F. mosseae*, *R. intraradices* and *Rhizophagus* species.

After eight months growing under tap water irrigation, a saline solution with 200 mM sodium chloride (NaCl) was applied, and an experimental assay established with four treatments: non-inoculated (NonInoc) and AMF-inoculated (Inoc) plants irrigated with tap water vs. Inoc and NonInoc plants irrigated with saline water. Five replicates of each treatment were performed for each species, and pot positions were changed each week. Plants were maintained under natural light, with average temperatures of 19.1 °C and relative humidity of 61.3% during winter, and 35.4 °C and 33.7% humidity during summer. The substrate electric conductivity (EC) showed an increase from 2.892 dS/m, in the beginning, to values ranging from 30.2 to 50 dS/m, by the end of the experiment, while the tap water-irrigated substrate showed values between 2.6 and 4.3 dS/m.

### 2.2. Stomatal Conductance and Chlorophyll Fluorescence

To evaluate plants physiological status and plant stress, foliar gas exchange (stomatal conductance to water vapour, g_sw_) and chlorophyll fluorescence parameters (quantum efficiency of photosystem II, ΦPSII) were recorded using a portable device LI-600 Porometer/Fluorometer (Licor 600, LI-COR Bio Sciences, Lincoln, NE, USA). The ΦPSII measures the proportion of light energy captured by PSII that drives electron transport during the photosynthesis, being calculated using maximum and steady-state fluorescence yields in a light-adapted leaf [41]. Measurements were non-destructive and conducted on all plants at the beginning and end of the experiment: after two months, when salinity effects were already evident, and one year after salt application; at 12 pm, three readings were taken on three randomly selected young leaves per plant, chosen from the plant central core.

### 2.3. Ultrastructure of Leaf Epidermal Cells

For the structural analysis of leaf tissue, epidermal imprints from three plants of each species and treatment (species × irrigation water × AMF inoculation) were prepared from the abaxial and adaxial surfaces of young fully expanded leaves. The imprints were prepared by applying a thin coat of transparent nail polish to the leaf surface; after drying, the resulting film was gently lifted using clear adhesive tape and affixed to microscope slides, according with [42]. Then, they were fixed on a microscope slide and examined under a Zeiss Axioskop microscope, using 100× magnification. Digital images of five randomly selected fields per imprint were captured using a Zeiss AxioCam digital camera (AxioCamHRm, ZEISS, Oberkochen, Germany), and the digital images were processed with Photoshop (Adobe Systems). The quantity and surface area (mm^2^) of the distinct cell types, specifically salt glands, stomata, and pavement cells, were determined using ImageJ software (version 1.49s). The epidermal leaf area occupied by each cell type, as well as the mean sizes of individual stomata and salt glands, were calculated (mm^2^). In addition, salt gland density was expressed as the number of salt glands per mm^2^ of leaf area.

### 2.4. Quantitative qRT-PCR

Young leaves from all treated plants were collected after one year of growth under saline conditions, immediately flash-frozen in liquid nitrogen, and stored at −80 °C for subsequent gene expression analysis using real-time quantitative PCR (qRT-PCR). Total RNA was extracted using the Analytik-Jena InnuSPEED Plant RNA Kit (Analytik Jena Innuscreen GmbH, Jena, Germany) [43]. RNA concentration and purity were assessed using a NanoDrop spectrophotometer (Thermo Scientific, Waltham, MA, USA), and integrity was confirmed by agarose gel electrophoresis. Only samples with A260/A280 ratios between 1.9 and 2.1 and clear 18S and 28S rRNA bands were used for downstream analysis. A total of seven genes were selected for analysis. The genes included *AtSOS1*: SALT OVERLY SENSITIVE1, an Na^+^/H^+^antiporter [44]; *AtP5CS1*: Δ1-PYRROLINE-5 CARBOXYLATE SYNTHASE 1, a proline biosynthetic gene [45]; *AtGSTU5:* GST CLASS TAU5, a glutathione antioxidant-related gene involved in stress resistance [45]; *LbTRY*: TRIPTYCHON, MYB protein (R3MYB-type) with high homology to TRIPTYCHON (*TRY*) of *Arabidopsis* which participates in trichome differentiation [37,44]; *Lb7G34824*: Lb7G34824 protein involved in salt gland development; *LbGIS2*: GLABROUS INFLORESCENCE STEMS 2 involved in cytokinin signal pathway [37]; and *TIP5*: tonoplast intrinsic protein 5;1, an aquaporin that functions as water and urea channels in pollen, regulated in *Limonium* species [46]. In this last gene, primer3 web version 4.1.0 [47] was used to design the primers with an e-value < 2 × 10^−4^ and a score > 41. All primer sequences are presented in Table S1. cDNA was synthesized from 1 μg of total RNA using the SensiFASTTM cDNA Synthesis kit (Meridian BioScience, Cincinnati, OH, USA), according to the manufacturer’s recommendations. PCR reactions were prepared using the SensiFASTTM SYBR No-ROX kit (Meridian BioScience, USA) following the manufacturer protocol. The amplification procedure was as follows: 94 °C for 5 min and 35 cycles of 30 s at 94 °C, 1 min at 51 °C, and 1 min at 72 °C. Relative expression levels were calculated using the formula 2^−ΔΔC(T)^. *Lbtubulin* was used as the internal reference (amplified with primers *LbTubulin_S and LbTublin_A*) for normalization following [44]. Its stability was supported both by this previous study and by our own dataset, where Ct values remained consistent across treatments (median CV = 1.3%), indicating minimal variability and suitability for use as a reference gene. Three biological replicates were used per treatment and species. Each biological replicate was analyzed in triplicate technical qRT-PCR reactions.

### 2.5. Statistical Analysis

Statistical analyses were performed using statistical software R Studio version 4.4.0 for Windows. For each species, a two-way analysis of variance (ANOVA) with interaction was applied to evaluate the effects of (i) arbuscular mycorrhizal fungi (AMF) inoculation and (ii) saline water irrigation treatment. Additionally, a *t*-test was performed on gene expression data to test the effect of species within each treatment. In all cases, data normality and homogeneity of variances were tested prior to the analyses. Square root or logarithm transformations were applied when necessary to meet ANOVA assumptions of homogeneity of variances and normality of residuals. When statistically significant (*p* < 0.05) differences were detected, pairwise comparisons were performed using a Tukey HSD post hoc test.

## 3. Results

### 3.1. Physiological Status

**Figure 1:**
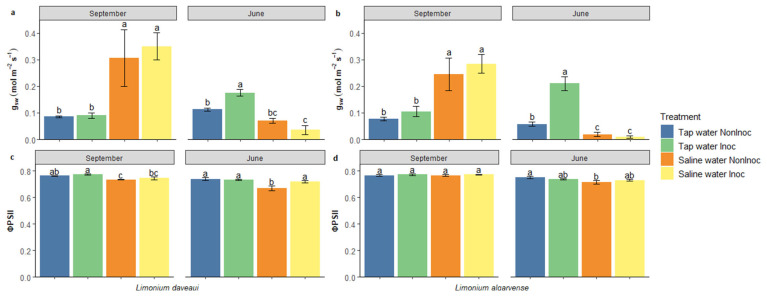
Physiological parameters measured in *L. daveaui* (**a**,**c**) and *L. algarvense* (**b**,**d**) at the beginning and end of the experiment, after two (September) and twelve (June) months of exposure to saline water irrigation, respectively. (**a**,**b**) Stomatal conductance (g_sw_; mol m^−2^ s^−1^); (**c**,**d**) Quantum efficiency of photosystem II (ΦPSII). Plants were inoculated or not with the halotolerant AMF consortium and irrigated or not with a saline solution (200 mM NaCl). Bars indicate the mean value per treatment ± standard error. Different letters at each time point indicate statistically significant differences according to the Tukey multiple comparison test. Abbreviations: NonInoc—non-inoculated plants; Inoc—AMF-inoculated plants.

Considering stomatal conductance to water vapour (g_sw_; Figure 1a,b), two months after saline water irrigation no differences were found between inoculated and non-inoculated plants. On the other hand, the irrigation treatment significantly affected this parameter in both studied species (*p <* 0.001). Quantum efficiency of photosystem II (ΦPSII) parameter also revealed a significant effect of irrigation in *L. daveaui* (*p <* 0.001) but no differences were found in *L. algarvense* values (Figure 1c,d).

In the beginning of the experiment both non-inoculated and inoculated plants in saline conditions had higher g_sw_ values than those in tap water. However, by the end of the experiment a shift in plants responses occurred among experimental groups and plants in saline conditions had the lowest values (Figure 1a,b). In *L. daveaui* plants, g_sw_ values differ significantly across the irrigation treatment (*p <* 0.001) and a significant interaction (*p <* 0.01) between factors was evident. As for *L. algarvense*, there was a significant effect of irrigation (*p <* 0.001) and AMF inoculation (*p <* 0.01) treatments as well as their interaction (*p <* 0.001). AMF inoculated plants had significantly higher g_sw_ than non-inoculated plants in tap water. By the end of the experiment, the irrigation treatment also affected significantly the ΦPSII values in both *L. daveaui* (*p <* 0.01) and *L. algarvense* (*p <* 0.05), with a significant interaction between factors (*p <* 0.05) in the first species (Figure 1c,d). Overall, in both species the lowest ΦPSII values were measured in non-inoculated plants under salinity.

### 3.2. Leaf Cell Composition

**Figure 2:**
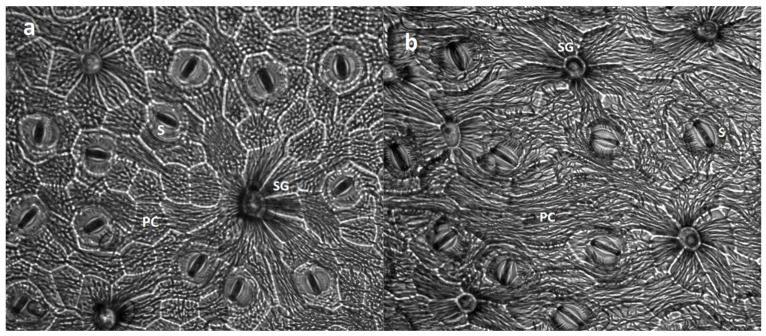
Adaxial epidermal cells of the studied *Limonium* species under salinity, with different AMF inoculation treatments. (**a**) Leaf epidermis of inoculated *L. daveaui* plants; (**b**) Leaf epidermis of non-inoculated *L. algarvense* plants. Scale Bar = 100 µm. Salt gland (SG), stomata (S) and pavement cells (PC).

**Figure 3:**
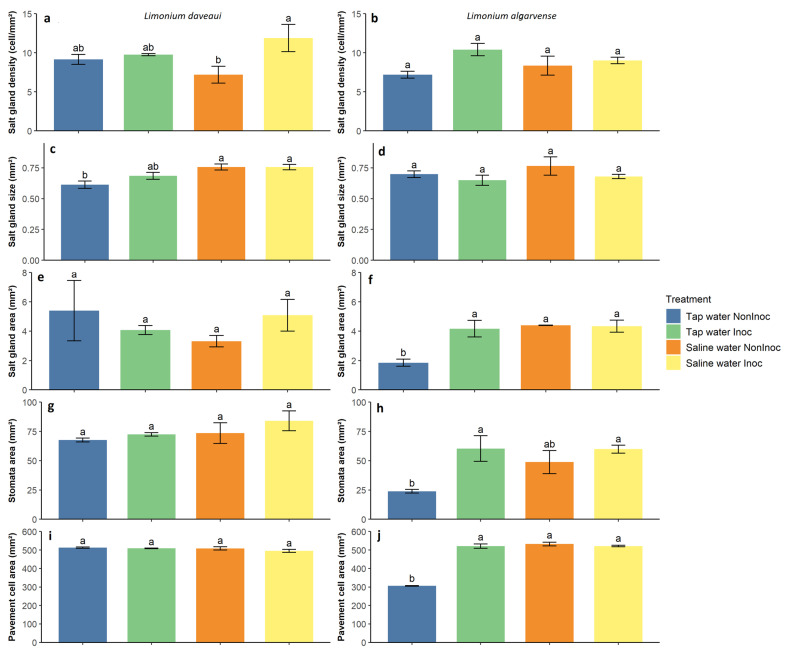
Leaf epidermal cell composition of *L. daveaui* (**a**,**c**,**e**,**g**,**i**) and *L. algarvense* (**b**,**d**,**f**,**h**,**j**) under the different experimental conditions studied. (**a**,**b**) Salt gland density; (**c**,**d**) Salt gland size; (**e**–**j**) Epidermal leaf area occupied by each cell type, specifically salt glands, stomata and pavement cells. Plants were inoculated or not with the halotolerant AMF consortium and irrigated or not with a saline solution (200 mM NaCl). Bars indicate the mean value per treatment ± standard error. Different letters at each time point indicate statistically significant differences according to the Tukey multiple comparison test. Bar plots without letters indicate that no significant differences were detected among treatments. Abbreviations: NonInoc—non-inoculated plants; Inoc—AMF-inoculated plants.

In the studied *Limonium* species, differences in leaf epidermal cell composition (Figure 2) were observed between and among the varying experimental treatments. Mycorrhizal inoculation influenced both species salt gland density (Figure 3a,b), with a significant increase in *L. daveaui* plants abaxial epidermis (*p* < 0.05) in saline water conditions. While, in *L. algarvense* salt gland density increased in the adaxial epidermis (*p* < 0.05) when irrigated with tap water. Regarding salt gland size, a significant effect of irrigation was noticeable in *L. daveaui* (*p* < 0.01), which had bigger glands under saline conditions than in tap water (Figure 3c). Although no differences were seen on the size of *L. algarvense* salt glands (Figure 3d), saline water irrigation and AMF inoculation significantly enhanced salt gland (*p* < 0.01; Figure 3f) and pavement cell areas (*p* < 0.001; Figure 3j), with a significant interaction between factors (*p* < 0.05; *p* < 0.001, respectively). Additionally, in *L. algarvense* plants, stomata area was also significantly increased in inoculated plants (F(1) = 14.459, *p* < 0.01; Figure 3h) whereas irrigation had no significant influence (F(1) = 5.297, *p* < 0.1). Contrastingly, these parameters revealed no differences in *L. daveaui* plants (Figure 3e,g,i).

### 3.3. Gene Expression

**Figure 4:**
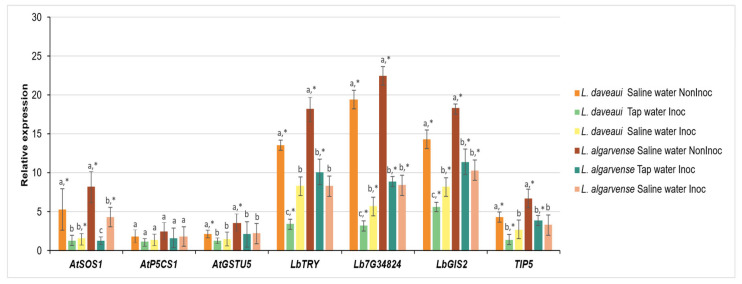
Expression levels of stress-related genes in *Limonium daveaui* and *L. algarvense*. Plants were inoculated or non-inoculated with the halotolerant AMF consortium and exposed to tap or saline water (200 mM NaCl) irrigation. Gene expression was quantified for seven genes, *AtSOS1*, *AtP5CS1*, *AtGSTU5*, *LbTRY*, *Lb7G34824*, *LbGIS2*, and *TIP5*. Bars represent mean ± standard error. Different letters above bars denote statistically significant differences between treatments within the same species while asterisks (*) indicate significant differences between species under the same treatment condition (*p* < 0.05).

The expression of seven salinity related genes was significantly modulated by salinity and AMF inoculation in both species (Figure 4). In general, genes were strongly upregulated under saline conditions, particularly in non-inoculated plants, although the magnitude and pattern of these responses varied between species and genes. For instance, in non-inoculated plants exposure to saline water triggered a sharp increase in the expression of *LbTRY* (~13-fold), *Lb7G34824* (~19-fold), and *LbGIS21* (~15-fold) in *L. daveaui*. *Limonium algarvense* showed the same trend, with even stronger responses, especially for *Lb7G34824*, which reached up to ~23-fold expression in the same conditions. By contrast, AMF-inoculated plants under saline water had a marked reduction in gene expression. For example, the expression of *Lb7G34824* dropped to nearly one-fourth of the levels observed in non-inoculated *L. daveaui* plants. Other genes, such as *AtSOS1* and *TIP5*, were expressed at more moderate levels (up to ~8-fold), but followed the same general pattern with the highest expression being recorded in non-inoculated plants irrigated with saline water. Notably, these genes had a reduced expression in AMF inoculated plants, with a higher level of expression in *L. algarvense*. The treatments had no significative effect on the expression levels of *AtP5CS1*.

## 4. Discussion

In this work, we analysed the influence of halotolerant arbuscular mycorrhizal fungi (AMF) inoculation on physiological status, leaf cell structure and expression of seven genes in two *Limonium* recretohalophytes grown under tap water or saline conditions. This study provides new insights into the physiological and molecular mechanisms by which AMF contribute to salinity tolerance in *Limonium* species. Our results show that AMF inoculation positively influences key physiological traits, such as stomatal conductance and photosystem II efficiency. Additionally, AMF-colonized plants under salinity exhibit reduced expression of genes involved in salt tolerance and in salt gland development. These improvements likely reflect enhanced water use efficiency and photosynthetic capacity, consistent with previous research on AMF-mediated stress alleviation in plants exposed to salt stress.

### 4.1. Halotolerant AMF Inoculation Enhances Stomatal Conductance and Photosynthesis

In this study, at the beginning of the experiment saline water irrigation had positive effects on both species leading to higher g_sw_ values than in plants maintained in tap water conditions (Figure 1a,b). An optimal salt concentration is beneficial for halophytes [8,13], promoting seedling growth, reflecting an improved photosynthetic efficiency [48,49]. In *Limonium* species leaf succulence increased at lower salinities after short term treatments with different NaCl concentrations or seawater (e.g., 0 and 10 dSm^−1^) but decreased at higher salinities [50,51]. The same pattern was observed for growth parameters in such species. In the studied *Limonium* species, salinity has been shown to positively affect photosynthetic indexes during the first months under 200 mM NaCl [39].

Stomata allow the exchange of gases between the leaf’s internal structure and the atmosphere, by controlling stomatal pore aperture [52,53]. Stomatal control is one of the earliest responses in water-deficit conditions, namely under drought and salt stress, together with transpiration rates reduction, photosynthesis and growth inhibition [2,54,55]. In *Limonium* plants, long-term salinity exposure significantly decreased both species g_sw_ and ΦPSII values (Figure 1). These effects are potentially related with the cumulative salinity levels evidenced by the high substrate EC measured at the end of the experiment. Stomata closure response could be an indicative of salt stress in *Limonium* plants maintained in saline conditions, resembling an “isohydric” pattern. A similar behaviour was previously reported for other *Limonium* spp. considered as drought-avoidant [56]. Isohydric plants respond to stress conditions by tightly regulating stomatal closure to minimize transpiration water loss, thereby maintaining elevated relative water content and preserving cellular hydration status [56,57].

Regarding the effect of AMF inoculation, significant differences were solely found by the end of the experiment, having inoculated plants significantly higher g_sw_ values in tap water (Figure 1a,b) and increased ΦPSII under salinity (Figure 1c,d). Positive results of AMF inoculation on g_sw_ values have also been observed in both crops (e.g., maize, rice) and non-crop species (e.g., *Robinia pseudoacacia*; halophyte *Asteriscus maritimus*) when cultivated in saline conditions, indicating a better plant water status [16,20,21]. Induced root proliferation and sturdier vascular systems in AMF plants are associated with increased transpiration rates and stomatal conductance [19]. In the salt and drought tolerant *Puccinellia tenuiflora*, mycorrhizal inoculation increased P and K concentrations, as well as reduced shoot Na accumulation, improving plant tolerance under the combined salt and drought stresses [58]. In this study, in AMF inoculated plants under salinity, ΦPSII increases could be related with a higher Rubisco specificity factor (relative capacity to catalyze carboxylation and oxygenation of ribulose 1,5 bisphosphate), as observed in other *Limonium* spp. in comparison with other C_3_ plants [56]. Thus, although by the end of the experiment g_sw_ values were lower in plants in saline conditions, AMF may have had a positive role in helping plants sustain a somewhat higher photosynthesis with stomata closed. Additionally, despite the varying effects of AMF inoculation across host plant species with different salt tolerant capabilities, AMF positive effects on halophytes growth promotion have been associated to energy efficient improvements in osmoregulation induced by inorganic ions and organic osmolytes [17].

### 4.2. AMF Inoculation Improves Salinity Tolerance by Altering Leaf Cell Composition

Salt glands play a critical role in salt tolerance by directly secreting the excess of salt onto the epidermal leaf surface [27,28]. This mechanism helps plants to maintain low internal salt levels, limiting salt damage and allowing them to withstand and adapting to high salinity habitats with improved photosynthesis and salt tolerance [59]. Salt gland density is controlled by several factors, including after short-term NaCl treatments at high concentrations as observed in the halophyte *Glaux maritima* [60] or the recretohalophyte *Limonium sinuatum* [61]. In *Limonium bicolor*, salinity tolerance is increased by enhancing salt gland density and photosynthesis [62]. In our study, saline water had a positive effect in both species, leading to bigger salt glands in *L. daveaui* (Figure 3c) and increasing salt gland and pavement cell areas in *L. algarvense* (Figure 3f,j). Similarly, in four other *Limonium* species, salt gland density and Na^+^ secretion were highly positively correlated with salt concentration [59].

Mycorrhizal inoculation influenced positively both species salt gland densities, potentially improving plant salt tolerance (Figure 3a,b). These changes support the enhanced g_sw_ and ΦPSII values observed in inoculated plants (Figure 1), highlighting the advantage of using halotolerant AMF.

Changes on salt gland density were seen in *L. daveaui* plants abaxial epidermis in saline conditions. Contrastingly, in *L. algarvense* salt gland density was increased in the adaxial epidermis of tap water irrigated plants. Moreover, AMF inoculation enhanced the areas of salt glands, stomata and pavement cells in *L. algarvense* grown under tap water (Figure 3f,h,j). This differential pattern seen among species indicates that other factors may be involved. For instances, different levels of salt tolerance have been related with differences in salt gland density observed among *Limonium* species [59]. Particularly, *Limonium* species grown in regions with highly saline soil have significantly higher salt gland density, thus, stronger salt secretion capacity and higher level of Na^+^ secreted outwards, than those growing in regions with little soil salinization [59]. In our case, both *Limonium* species thrive in saltmarshes although occupying different positions. *Limonium algarvense* thrives in the intertidal zone, while *L. daveaui* is on the margins of marsh slopes [34,35]. Since *Limonium daveaui* is only inundated during the equinoctial tides, higher salt concentrations might be accumulated on the soil profile [35]. These observations support the relation between species salt tolerance and their natural environment, potentially explaining changes seen in the anatomical structures of both species under different water irrigation regimes. Additionally, although no convincing evidence has been presented demonstrating that AMF are host-specific, host preferences and host selectivity have been widely reported [63], with differences in fungal behaviour or characteristics in interacting with hosts [64]. Here, the origin of the halotolerant AMF consortium isolated from the rhizosphere of saltmarsh *L. daveaui* plants can be associated to a species-specific effect.

### 4.3. Halotolerant AMF Inoculation Differentially Reduces the Expression of Salt-Related Genes

The gene expression patterns reported in our study reinforce the role of microbial inoculants as effective modulators of plant stress responses in recretohalophyte species. Both *Limonium daveaui* and *L. algarvense* showed a significant upregulation of selected genes under saline conditions (Figure 4), mostly in the absence of AMF inoculation, suggesting a strong activation of their endogenous protective mechanisms.

Particularly, genes which play a crucial role in regulating Na^+^ influx, and overall salt stress response, and membrane aquaporins were expressed at moderate levels with the highest expression being recorded in non-inoculated plants irrigated with saline water. The marked reduction in expression in inoculated plants under saline conditions supports the hypothesis that beneficial microbes may confer physiological resilience by either priming the plant’s defense system or by directly alleviating stress through enhanced nutrient uptake, hormonal modulation, or oxidative protection [65,66,67]. Moreover, our findings are consistent with previous reports demonstrating that endophytic microorganisms can suppress stress-induced gene expression while maintaining or even improving plant performance under saline or drought stress [68,69]. For instance, in lettuce plants the late embryogenesis abundant protein (*LsLea*), acting as a stress marker and having a protective role during osmotic stress, is express under salt stress conditions. However, the induction of this gene is lower in AMF plants than in non-inoculated plants [70]. The lower gene expression suggests that inoculated plants suffer less stress than non-AMF plants, likely due to primary salt avoidance mechanisms [19]. Moreover, in mycorrhizal inoculated trifoliate orange plants under salinity, the AMF mitigated the effects of salt stress by improving physiological responses and regulating the expression of root tonoplast intrinsic protein (*TIPs*) genes [25]. These membrane proteins (aquaporins) regulate water movement and help maintain osmotic adjustment under saline conditions.

Regarding genes found to be involved in salt gland differentiation, salt gland density, and salinity tolerance in *L. bicolor*, such as *LbTRY*, *Lb7G34824*, and *LbGIS2-Lb4G22721* [62], a detailed analysis of the gene regulatory network showed interesting results [37]. *LbTRY* was highly expressed in salt glands and interacted physically with *Lb7G34824* and both negatively regulated salt gland development and salt secretion by regulating the expression of cytokinin-related genes [37]. While, in the absence of *LbTRY*, relative low expression of some cytokinin-related genes will promote salt gland initiation. Moreover, in both *LbTRY*-silenced and *Lb7G34824*-silenced plants, compared with the controls, the expression of *LbGIS2* was substantially downregulated [37]. In our study, the reduced expression of these genes in AMF-inoculated plants, despite their increased salt tolerance, may reflect a more efficient or alternative mechanism of ionic regulation, and water balance and nutrient supply facilitated by mycorrhizae. Rather than relying on extensive salt gland differentiation and activity, AMF-colonized plants may benefit from improved root-shoot signaling, ion compartmentalization, and enhanced antioxidant protection, reducing the need to activate stress-inducible developmental pathways.

In both species AMF inoculated plants, the genes involved in salt gland differentiation showed a reduced expression, highlighting the protective role of the AMF consortium in enhancing salinity tolerance under prolonged salinity exposure. Interestingly, *L. algarvense* appeared to exhibit a more pronounced genetic activation under stress than *L. daveaui*, which may reflect a species-specific sensitivity or adaptive strategy. On the other hand, the protective effect of AMF inoculation is weaker in this species, possibly due to the use of a microbial consortium isolated from *L. daveaui*. This differential response highlights the importance of considering genotype-specific interactions in the design of bio-inoculant strategies.

## 5. Conclusions

This study demonstrates that inoculation with halotolerant arbuscular mycorrhizal fungi (AMF) significantly modulates the physiological and molecular responses of *Limonium* species under long-term salinity stress. AMF enhanced stomatal conductance, photosynthetic efficiency, and certain leaf epidermal traits, contributing to improved stress resilience.

Although initially salinity had positive effects on plant physiological status, after one year in saline conditions, an isohydric response was described for *L. daveaui* and *L. algarvense*. Nevertheless, inoculated plants had higher ΦPSII than the non-inoculated ones under salinity. Thus, pointing to a positive role of AMF in helping plants sustain higher photosynthesis with stomata closed. Besides, the effect of AMF inoculation on plant salt tolerance may be correlated with the increased density of salt glands, possibly improving plant’s ability to secrete Na^+^ outwards.

Notably, salinity and AMF treatments influenced the expression of key genes involved in salt tolerance, including *AtSOS1*, *TIP5*, and salt gland development-related genes (*LbTRY*, *Lb7G34824*, and *Lb4G22721GIS2*), with AMF inoculation generally reducing gene expression under salinity. Overall, the suppressed stress-induced gene expression by AMF could reduce the need to activate stress-inducible developmental pathways under highly stress conditions.

*Limonium algarvense* showed more pronounced changes in epidermal structure and gene expression levels compared to *L. daveaui*, suggesting varying degrees of dependence on or responsiveness to AMF symbiosis. The differential effects observed among the two *Limonium* species studied reflect a species-specific sensitivity or adaptive strategy, possibly related to each species natural environment and the different levels of salinity to which they are adapted. Besides, the origin of the halotolerant AMF consortium isolated from the rhizosphere of saltmarsh *L. daveaui* plants can be associated to a species-specific effect. These findings highlight the complex interplay between salinity, symbiotic fungi, and host plant genotype in shaping adaptive strategies. Overall, halotolerant AMF inoculation presents a promising approach to enhance salt tolerance in halophytes, with implications for sustainable cultivation in saline environments.

## Data Availability

The original contributions presented in this study are included in the article/Supplementary Materials. Further inquiries can be directed to the corresponding author(s).

## Supplementary Materials

The: following: supporting: information: can: be: downloaded. at: https://www.mdpi.com/article/10.3390/genes16091084/s1, Table S1: Primers used in this study for qRT-PCR.
