# Supplementary material for: Halotolerant Mycorrhizal Symbiosis Enhances Tolerance in Limonium Species Under Long-Term Salinity
Source: Genes (Basel). 2025 Sep 15;16(9):1084. doi: 10.3390/genes16091084 (PMC12470191; doi:10.3390/genes16091084)
Supplement: Supplementary file 1 [file genes-16-01084-s001.zip › genes-3822871-supplementary.pdf]

Supplementary Materials

Table S1. List of gene-specific primers used for qRT-PCR analysis in this study, including forward (S) and reverse (A) sequences. LbTubulin served as the internal reference gene. E: efficiency values for each gene. Ct: median *Ct*-values and coefficient of variation (% between brackets).

**Gene symbol**	**Primer sequence (5′–3′)**	**E**	**Ct**
AtSOS1_S	TTCATCATCCTCACAATGGCTCTAA	96.8	6.01 (1.8)
AtSOS1_A	CCCTCATCAAGCATCTCCCAGTA		
AtP5CS1_S	CAAGATGAGATTACATTCG	97.3	4.35 (2.1)
AtP5CS1_A	GGTTATGATGACAGGAAT		
AtGSTU5_S	ATGGCTGAGAAAGAAGAAGTGAAGC	98.4	5.54 (2.5)
AtGSTU5_A	TTAAGAAGATCTCACTCTCTCTGCC		
*LbTubulin_S**	GGTTGAGTGAGCAGTTCAC	99.6	4.02 (1.3)
*LbTubulin_A**	GATAACCAGCCACACCTTAGC		
LbTRY-S	CTGAGGAAATTGAGAGGTTT	96.2	5.42 (2.7)
LbTRY-A	CTATCATCATCACGGTTGTTA		
Lb7G34824-S	CACAGTAGGAATGATAACC	98.3	6.15 (1.8)
Lb7G34824-A	GTGAGTGACTAACAGAGA		
LbGIS2-S	CTCCAATCTCGTTACTAATC	97.4	5.34 (1.9)
LbGIS2-A	ATCTTCTGGGTTGACTTC		
TIP5-S-S	GGCATCAACAGGGACAACAG	96.6	6.01 (2.1)
TIP5-S-A	TAAAGCTCCCTCCAATGGCA		

*internal reference
